# Addition of *Spirulina platensis* to Laying Hens’ Diet: Impact on the Egg Quality and Oxidative Stress Biomarkers

**DOI:** 10.3390/life16081296

**Published:** 2026-08-06

**Authors:** Christos Eliopoulos, Ioanna Langousi, Matthaios Tselios, Eleni Kougia, Vasileios G. Papatsiros, Giorgos Markou, Dimitrios Arapoglou

**Affiliations:** 1 Institute of Technology of Agricultural Products, Hellenic Agricultural Organization-Demeter (HAO-Demeter), 14123 Athens, Greece; ilagousi@gmail.com (I.L.); mattblin457@gmail.com (M.T.); elenikougia8@gmail.com (E.K.); markougior@elgo.gr (G.M.); 2 Clinic of Medicine, Faculty of Veterinary Science, University of Thessaly, 43100 Karditsa, Greece; vpapatsiros@vet.uth.gr

**Keywords:** *Spirulina platensis*, oxidative stress, laying hens, productive performance, yolk color, egg weight, microalgae

## Abstract

The present study examined the supplementation of *Spirulina platensis* (SP) in various ratios in the basal diet of laying hens targeting the determination of the optimum amount that is required for the improvement of egg quality parameters and oxidative stress. A total of 300 24-week-old Lohmann Brown laying hens were selected and kept at a commercial farm (Vasileios Kompoulis Ltd. farm, Megara, Attiki, Greece). The employed farm was supplied with 25 similar cages. The control group was fed the basal diet, whereas the experimental feedstuff was fortified with SP by 0.1, 0.2, 2 and 5% *w*/*w*. At the end of the trial the addition by 5% *w*/*w* improved the egg weight, the yolk color, carotenoids, protein content and enhanced the eggshell strength. Additionally, the specific treatment improved the oxidative stress of the employed laying hens by recording the lowest values in TBARS and CARBs content. On the contrary the fortification with 5% *w*/*w* enhanced TAC, GSH and CAT activity. Overall, SP addition forms an intriguing approach for the improvement of the performance, physiology, and health of laying hens, with a parallel contribution to the development of more sustainable and viable feed ingredients for poultry production systems.

## 1. Introduction

Animals’ health and productivity are mainly affected by the employed feedstuffs, with a parallel impact on the generated livestock products. Currently, there is a trend that focuses on the nutritional upgrade of the applied animal feed by incorporating various bioactive compounds targeting the enhancement of animals’ welfare [1]. It must be noted that according to a plethora of studies in the existing literature this strategy has led to very promising results since it is closely associated with the improved status of animals’ productivity and welfare [2].

Poultry farming is considered one of the most significant industries that can meet the global need for animal proteins. Additionally, the latter is very susceptible to oxidative stress as well as to a plethora of diseases that can directly impair its health and productivity. Therefore, this fact necessitates the development of sustainable solutions in order to address this issue, with the employment of some natural compounds forming an intriguing option, even though conventional diets meet their nutritional needs [2,3,4].

Oxidative stress forms a dangerous threat for laying hens, especially during the production period. This condition can lead to serious pathological conditions including infections, heat stress, etc. [5]. Therefore, there is a need to develop novel sustainable and viable practices, such as the adoption of antioxidant compounds, in order to address the production of free radicals [6,7].

Nowadays, algae such as *Spirulina platensis* (SP), which are characterized as natural ingredients, have gained interest mainly due to their physicochemical properties. Algae are characterized as photosynthetic organisms and they are located in aquatic systems. Their structure varies from single-celled microalgae to large seaweeds [4,8]. *Arthrospira platensis* is a filamentous blue-green algae [9,10,11] and is usually called Spirulina (SP). It is classified in the group of Cyanophyta/Cyanobacteria, which are found in natural warm and alkaline aquatic environments. Furthermore, SP can be characterized as a functional food and is considered safe (GRAS) by the European Food Safety Authority (EFSA) [12]. This can be rationalized by SP’s rich chemical profile, since the latter is highlighted by the presence of high protein content, essential amino acids, vitamins, minerals and various bioactive compounds [13,14]. Additionally, SP forms an ideal feed additive material since its physicochemical composition contains significant ingredients, namely vitamin B12 γ-linolenic fatty acid, β-carotene, calcium, and iron [4,13,14]. Vitamin B12 is responsible for the production of red blood cells and immune system performance, while γ-linolenic fatty acid promotes the preservation of cell membrane’s integrity. β-carotene is considered as a significant compound facilitating vision, immune activity and skin health [15]. Calcium’s presence is of high importance since it is essential for bone health securing thus the ability for the proper performance of the skeletal system, while iron’s concentration is responsible for oxygen’s transfer in blood, with a parallel contribution in metabolic activity [16,17]. Additionally, SP’s existing compounds contain several bioactive molecules that exert positive health-promoting effects. More specifically, SP’s nutritional content is characterized by the presence of phycocyanin. Phycocyanin is a molecule with a strong antioxidant and anti-inflammatory profile, protecting the cells by scavenging free radicals, thus preventing the development of oxidative stress [18]. Additionally, the included polysaccharides in SP’s composition present a similar pattern with phycocyanin concerning the beneficial health effects. Specifically, their main activity focuses on the improvement of the immune system and natural killer cells, as well as on the enhancement of macrophages’ activity [4,19]. Finally, it must be noted that the combination of the latter bioactive molecules could presumably exert a positive impact in poultry sector indicating SP as an ideal feed supplement, highlighting its potential to enhance their immune system and mitigate the generated oxidative stress, thus promoting laying hens’ welfare and productivity. *Spirulina platensis* has been widely used in the poultry nutrition both in low and high concentrations. SP’s inclusion in low levels has been investigated as a functional feed additive where the beneficial impact can be rationalized presumably by SP’s antioxidant, immunomodulatory and bioactive properties. On the other hand, SP’s presence in high amounts has been evaluated as a nutritional material since it enhances the prepared diet with protein, essential amino acids, lipids, vitamins, minerals and other nutrients, which may affect animal’s performance in addition to its functional properties. Therefore, the obtained results at higher dietary inclusion levels should consider both the biological activity and the nutritional contribution of Spirulina.

Compared with previous reports, which have generally examined SP’s supplementation within a limited inclusion range (typically ≤ 2.5% *w*/*w*) [11,20,21,22,23], the present study evaluates a broad SP addition across a micro-additive/functional range (0.1 and 0.2% *w*/*w*) and a macro-ingredient range (2 and 5% *w*/*w*) within the same trial, combined with a multi-biomarker antioxidant panel (TBARS, protein carbonyls, TAC, GSH and CAT) and detailed egg fatty acid profiling. This design provides novel comparative insight into how the dose of SP affects the egg quality and the oxidative stress status of laying hens, as well as the relative contribution of SP’s functional bioactive compounds versus its nutrient dilution/nutrient density effects at higher inclusion levels.

The main aim of this study concerned the determination of the optimum SP addition in laying hens’ basal diet targeting the improvement of the quality of the produced eggs such as Haugh unit score, eggshell strength, thickness, and yolk color. The final goal was to evaluate the impact of SP addition on some antioxidant biomarkers that are directly connected with oxidative stress.

## 2. Materials and Methods

### 2.1. Animals, Diets and Experimental Design

A total of 300 24-week-old Lohmann Brown laying hens were selected and kept at a commercial farm (Vasileios Kompoulis Ltd. farm, Megara, Attiki, Greece). The employed farm was supplied with 25 similar cages. All groups had ad libitum access to feed and water during the trial. The rearing experiment included a control group (C) and four experimental treatments that were divided according to SP’s addition by adding 0.1% (SP0.1), 0.2% (SP0.2), 2% (SP2), and 5% (SP5) *w*/*w* to the conventional feedstuff. Each trial was performed in quintuplicate, and every cage was composed of 12 laying hens. At the beginning of the experimental procedure the average initial body weight was monitored at 1.503 ± 0.097 kg. All the employed birds were treated according to the European Union guidelines that concern the promotion of health and welfare, which should be followed for scientific procedures [24]. The rearing trial lasted for 60 days. Feed was provided on a weekly basis.

SP’s inclusion in the experimental diets was applied “on top” of the basal diet instead of replacing some ingredients. Consequently, the experimental diets were not formulated to be iso-nitrogenous or iso-caloric. Therefore, the final diet weights differed among treatments according to the amount of Spirulina added. The ingredient composition of the basal diet and the calculated nutrient composition of the final experimental diets are presented in Table 1. The four experimental diets were employed in order to examine SP’s effect as a micro- and macro-ingredient addition on laying hens’ performance, egg quality and oxidative stress status either as a result of its bioactive molecules or the combination of nutritional and functional contribution. Under the employed commercial rearing conditions in the present trial, oxidative stress was considered as a physiological consequence of intense metabolic and biosynthetic demands of egg production in laying hens, rather than the result of an externally applied experimental stressor (e.g., a controlled heat stress or nutrient restriction model); no additional stress challenge beyond standard commercial housing and management conditions was applied to the birds.

A commercial powder of *Spirulina plantensis* Strain SAG 257.80 (Spirulina Supreme, Spirulina Maxima I.K.E., Serres, Greece) was used in the present study. The specific microalga was cultivated in Greece under controlled greenhouse conditions and consisted of 100% *Spirulina* algae.

### 2.2. Analyses

#### 2.2.1. Determination of Egg Quality Parameters

The parameters that concerned the eggs’ external and internal quality characteristics were assessed. The external characteristics of the eggs (weight and diameter) were determined using a digital caliper (0.01 mm) and a digital scale, respectively. The latter procedure was followed by the egg breaking in order to separate the egg content of yolk and albumen. The eggshell was carefully washed and dried overnight at 80 °C. Subsequently, the eggshell weight was measured by employing a digital scale, while thickness was evaluated by using a digital caliper (0.01 mm) [25]. Additionally, the assessment of the internal quality attributes, namely yolk color, yolk and albumen height, diameter, weight, moisture content and pH were investigated. Yolk color was investigated by using the 16-point color scale fan of DSM-Firmenich YolkFan whereas egg’s weight was examined by using a digital scale. Yolk and albumen height were determined with a digital caliper (0.01 mm), their pH values were assessed according to an electronic pH meter while moisture content was evaluated after drying the samples at 100 °C for 24 h [26,27]. Additionally, the Haugh unit was calculated by using the formula described by Kul and Seker [28]—HU = 100 log (albumen height (mm) + 7.57 − 1.7 W^0.37^), where W = egg weight (g)—whereas Yolk Index was determined by applying the following equation—Yolk Index = Yolk Height/W—where h is the height and w the width both in mm [29]. Protein content was examined by applying the Kjeldahl method, which is used for total nitrogen evaluation. The protein concentration was calculated by multiplying the determined nitrogen content (Kjeldahl method) by the factor of 6.25 [30]. Finally, the egg strength was evaluated by using the Stable Micro Systems apparatus. Three eggs were randomly selected from each cage at the end of the experimental period (Day 60).

#### 2.2.2. Egg Yolk Total Carotenoid Content

Total carotenoid content was determined in freeze-dried egg yolk samples by applying a slightly modified version of the method described by Muhammad et al. [31]. Briefly, 0.05 g of fresh yolk was mixed properly with 1 mL of acetone. The solution was vortexed until the development of a homogenous mixture. Subsequently, 0.1 mL of distilled water and 0.5 mL of n-hexane were added in the homogenized sample followed by a centrifugation at 3000 *g* for 10 min. Finally, the n-hexane layer was collected. Pigmentation was measured (n-hexane layer) spectrophotometrically at 450 nm and was calculated according to the following equation:

$$\mathrm{Concentration}\;(\mathrm{mg}/g)=-\frac{Absorbance\times Volume\left(mL\right)\times 10,000}{A\;10\%\;1\;cm\times sample\;weight}$$

where A 10% 1 cm = 2592 for beta carotene as a constant.

#### 2.2.3. Fatty Acid Profile Determination

Fatty acid profile determination was performed by applying gas chromatography (Shimadzu Nexis GC-2030, Shimadzu Corporation, Kyoto, Japan) equipped with a dielectric barrier discharge ionization detector and a capillary column Mega-10 (0.25 mm diameter, 0.25 μm particle size, 60 m length). The initial temperature of the method was adjusted at 60 °C with an increase of 2 °C per minute, while the detector’s and split’s temperature was set at 245 °C. The extraction procedure was performed on freeze-dried egg yolk samples. In specific, 0.2 mg of sample were mixed with 10 mL of a chloroform:methanol solution in a 2:1 ratio followed by an addition of 2 mL of NaCl-saturated water leading to a phase separation. The chloroform layer from the previous step was collected and evaporated under nitrogen gas conditions. Subsequently, the samples were esterified by adding 1 mL heptane and 100 μL of an ethanolic KOH solution and incubated for 10 min at room temperature. Finally, the samples were filtered with a 0.45 μm syringe filter and then were subjected for GC analysis [32].

#### 2.2.4. Blood Sampling

At the end of the trial (60 days post-trial initiation) 15 blood samples were collected from three randomly selected birds per cage recording a total number of 75 samples. The employed birds were manually restrained and a total volume of 2.5 mL of blood sample was drawn from the wing vein. The collected blood samples were placed in BD Vacutainer plasma tubes which acted as anticoagulant agents. Subsequently, all the recovered samples were centrifuged at 12,000× *g* for 10 min at 4 °C. At the end of the process plasma was collected in order to evaluate total antioxidant capacity (TAC), Thiobarbituric acid–reactive substances (TBARS) and protein carbonyls (CARBs). The packed erythrocytes were lysed by adding distilled water at a ratio of 1:1 *v*/*v* followed by vigorous stirring. Subsequently, the samples were centrifuged at 4020 *g* for 15 min at 4 °C. The supernatant that represented the erythrocyte lysate was collected for the determination of GSH and catalase activity.

#### 2.2.5. Oxidative Stress Biomarker Assessment

Thiobarbituric acid–reactive substances (TBARS) were examined according to a modified version of the Keles et al.’s [33] method. The TBARS content (μmol/L plasma) was estimated according to molar extinction coefficient of malondialdehyde (MDA). Protein carbonyls (CARBs) were assessed by applying a method described by Patsoukis et al. [34]. Specifically, the CARB content was calculated and quantified according to the molar extinction coefficient of 2,4-Dinitrophenylhydrazine (DNPH). The results were expressed as nmol/mg protein. Total antioxidant content (TAC) was determined by performing the protocol of Janaszewska and Bartosz [35]. The obtained results were described as mmol DPPH/L plasma. Reduced glutathione (GSH) was measured according to the methodology that stated by Ready et al. [36]. GSH’s results were expressed as μmol/g Hb. Finally, catalase activity (CAT) was evaluated by applying the method described by Aebi [37]. Assay’s calculation was carried out according to the molar extinction coefficient of H_2_O_2_. All the analyzes were performed in triplicate.

#### 2.2.6. Statistical Analysis

All analyses were performed in triplicate and the results were expressed as mean ± standard deviation. Data normality was evaluated using the Shapiro–Wilk test. Differences between the examined parameters were evaluated by applying one-way ANOVA followed by Tukey’s post hoc test for multiplicity with significance set at *p* < 0.05.

## 3. Results

Table 1 presents the ingredients and the chemical composition of the employed diets during the trial period. The basal diet formulation was maintained across the employed treatments since SP was added on top at 0.1, 0.2, 2 and 5% per 100 g of the basal diet. The final mixtures of the generated feedstuffs were 100.1, 100.2, 102 and 105%, respectively.

**Table 1:** Ingredients and chemical composition of diets.

Ingredients/Calculated Nutrients	Control	SP0.1	SP0.2	SP2	SP5
Corn	40.00	40.00	40.00	40.00	40.00
Wheat bran	22.49	22.49	22.49	22.49	22.49
Soybean meal	24.36	24.36	24.36	24.36	24.36
Vegetable oil	1.48	1.48	1.48	1.48	1.48
DL-Methionine	0.16	0.16	0.16	0.16	0.16
Calcium carbonate	8.83	8.83	8.83	8.83	8.83
Monocalcium phosphate	1.32	1.32	1.32	1.32	1.32
Salt	0.33	0.33	0.33	0.33	0.33
Choline premix	0.04	0.04	0.04	0.04	0.04
Vitamin–mineral premix	1.00	1.00	1.00	1.00	1.00
Basal diet (parts)	100.00	100.00	100.00	100.00	100.00
*Spirulina platensis* added on top (parts per 100 parts basal diet)	0.00	0.10	0.20	2.00	5.00
Final mixture weight (parts)	100.00	100.10	100.20	102.00	105.00
Actual Spirulina concentration in final mixture (%)	0.00	0.10	0.20	1.96	4.76
Calculated nutrient composition of the final mixtures					
Metabolizable energy (kcal/kg)	2750	2751	2751	2761	2776
Crude protein (%)	17.00	17.04	17.08	17.83	19.02
Lysine (%)	0.870	0.872	0.873	0.903	0.949
Methionine + cystine (%)	0.730	0.731	0.732	0.752	0.783
Threonine (%)	0.640	0.642	0.644	0.680	0.736
Calcium (%)	3.90	3.90	3.89	3.83	3.72
Total phosphorus (%)	0.63	0.63	0.63	0.63	0.63

SP: *Spiroulina platensis*.

Table 2 presents the chemical composition of the employed SP that was used in the present study. Specifically, SP was composed of a high crude protein content of 67.1%, 8.2% lipids, 11.6% carbohydrates, 3.8% dietary fiber and 8.05% ash. Additionally, its profile contained 2.36% phycocyanin, 143 mg/kg β-carotene and B-group vitamins.

Table 3 presents the physicochemical characteristics of the pre-trial values that characterize the egg population at the beginning of the experiment (baseline reference).

Specifically, the weight of the control egg sample was 47.45 g, the height 51.90 mm and the diameter 40.44 mm. Regarding yolk characteristics, the weight recorded a value of 8.65 g, whereas moisture content was 51.34%. Yolk’s height and diameter were found to be 11.39 mm and 33.68 mm, respectively. The color recorded a score of 11.00 in the respective color fan. Albumen’s weight and height reached 28.32 g and 5.83 mm, respectively, moisture content was 86.92%, while pH parameter showed a value of 9.73. Carotenoid concentration was estimated at 64.83 mg/g, whereas the corresponding values of the eggshell thickness and weight reached 0.40 mm and 4.11 g, respectively.

Table 4 presents the results that concern the external and internal egg quality characteristics of the examined eggs at Day 60 for control and all SP additions. Specifically, in terms of the external quality parameters, the weight, height and diameter were found to be almost identical among the examined treatments. The SP5 addition recorded a statistically significant increase (*p* < 0.05) compared to the other samples, with the corresponding values reaching 73.15 g, 60.65 mm and 50.12 mm, respectively. Eggshell thickness and weight followed a similar pattern, since the SP5 addition revealed a statistically significant increment (*p* < 0.05) for both parameters at the end of the trial (0.69 mm and 8.49 g, respectively).

The internal egg quality attributes presented a similar behavior. In specific, after the SP5 treatment, the parameters of yolk weight, height and diameter were found to be significantly increased (*p* < 0.05), while the latter treatment simultaneously recorded the highest values of 18.78 g, 17.73 mm and 40.48 mm, respectively at Day 60. The characteristics that related to yolk’s moisture content and pH remained almost intact for all the examined samples at the end of the trial. On the other hand, SP5 addition presented a significantly increased content (*p* < 0.05) compared to the other samples at the end of the trial, with the corresponding values reaching 14.20 and 0.51, respectively.

**Figure 1:**
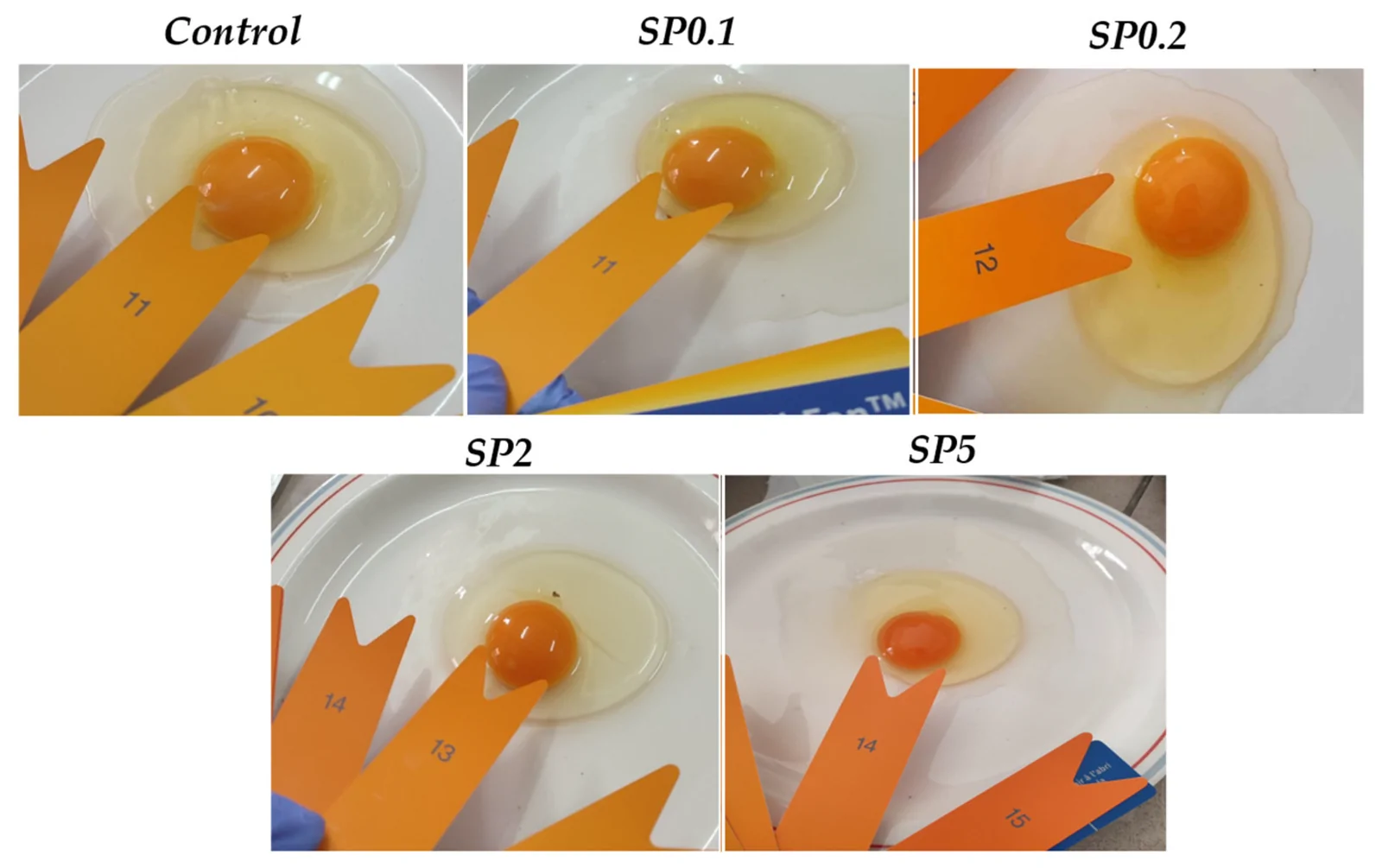
Effect of various addition levels of *Arthrospira platensis* on egg yolk color scores evaluated by the DSM Yolk Color Fan.

Figure 1 depicts the effects of the various addition levels of *Arthrospira platensis* on egg yolk scores that were assessed using the DSM Yolk Color Fan. The comparison of the yolk color between the examined treatments indicates the fact that SP5 addition enhanced the yolk color at Day 60.

Albumen’s characteristics presented an identical trend. In particular, the obtained results of SP5 treatment regarding albumen’s weight and height were found to be significantly increased (*p* < 0.05) compared to the other treatments, with the respective values reaching 46.01 g and 10.46 mm on Day 60. Albumen’s moisture and pH levels did not vary during the experimental period for all the examined treatments. Finally, the Haugh unit and Yolk Index exhibited a significant increment (*p* < 0.05) with the corresponding values reaching 76.22 and 0.51, respectively, on Day 60 for SP5 addition across all the investigated samples.

In the similar vein egg strength values were found to be almost identical for the control sample and the SP0.1, SP0.2 and SP2 addition. The SP5 sample recorded a statistically significant increment (*p* < 0.05) among the examined additions, with the respective value reaching 5.09% at the end of the trial.

According to Table 5 carotenoid content revealed a gradual increase between the investigated samples with the SP5 addition revealing the highest concentration of 172.83 mg/g (*p* < 0.05). Yolk protein content was found to be similar among almost all the examined samples apart from those with the SP5 addition, since the latter displayed a significant increase with the respective value reaching 40.84%. On the other hand, albumen proteins levels were almost intact in the control sample and the SP0.1 and SP0.2 trials, whereas SP2’s and SP5’s presence increased protein content significantly, with the corresponding values being 90.77 and 93.39% (*p* < 0.05). Finally, eggshell ash content was found to be almost identical across all the examined treatments.

Table 6 summarizes the results of the fatty acid composition of the investigated treatments at the end of the trial. Specifically, oleic acid (C18:1 Cis 9) and palmitic acid (C16:0) were found to be the major fatty acids, with their content ranging from 37 to 40% and 16–28%, respectively. It was observed that SP’s addition did not affect their content significantly. It must be noted that stearic acid recorded a statistically significant increase (*p* < 0.05) between the control sample and the enriched additions. In particular, stearic acid recorded a value of 7.87% for control, whereas it was found to increase (*p* < 0.05) for all SP additions of 0.1, 0.2, 2 and 5%, with the corresponding values reaching 9.22, 8.64, 9.27 and 8.84, respectively. Unlike heptadecanoic acid (C17:0), a-linoleic acid (C18:3 Cis 3) displayed a minor decreasing trend with the obtained values fluctuating closer to control levels as SP enrichment reached the level of 5% *w*/*w*. Total monosaturated fatty acid presence was stable across all the investigated samples, while total polyunsaturated fatty acids were significantly decreased in the fortified samples compared to control.

Table 7 presents the results that concern the effect of SP’s additions on laying hens’ oxidative profile. In specific, TBARS were found to be significantly reduced (*p* < 0.05) between the examined samples with the SP5 addition revealing the lowest value (10.34 μmol/L plasma). A similar behavior was observed for CARBs’ presence, since latter recorded a gradual reduction among the trials with SP5 addition displaying the highest statistically significant (*p* < 0.05) decrease since the corresponding value reached 0.54 nmol/mg protein. Conversely, TAC was found to be increased for all SP additions compared to the control sample, with SP5 presenting the highest statistically significant increment (*p* < 0.05) with the respective score reaching 1.23 mmol DPPH/L plasma. GSH levels displayed a significant increment (*p* < 0.05) across the investigated treatments, with SP5 addition revealing the highest content of 31.19 μmol/g Hb. Finally, CAT followed an identical pattern, since its activity was gradually increased between the examined trials, with the addition of 5% *w*/*w* indicating the highest concentration and the respective value reaching 25.69 U/mg Hb (*p* < 0.05).

## 4. Discussion

The present study investigated the effects of SP addition at various levels in laying hens’ diet, targeting the evaluation of its impact on egg quality parameters and laying hens’ antioxidant profile. The obtained results indicated that the supplementation in low amounts did not affect significantly the investigated characteristics. On the other hand, the increased presence of SP in laying hens’ diet exerted a significant impact on egg quality parameters (external and internal), as well as on their antioxidant profile.

According to the results, the weight of the produced eggs was almost identical across almost all the examined treatments. However, the SP5 addition displayed a beneficial effect, since the weight was found to be increased at the end of the experimental trial. This fact can be justified by the gradual impact of SP’s rich nutritional content, which is characterized by high of protein content, essential amino acids, vitamins, minerals, as well as a plethora of bioactive molecules, including β-carotene and γ-linolenic acid [4,13,14]. Another possible explanation could be associated with the presence of nutrients in SP’s nutritional profile. Specifically, it may have the potential to stimulate the ovaries, facilitating the production of larger eggs. Additionally, algae’s diverse benefits, such as their antioxidant and antimicrobial properties, may exhibit a positive impact concerning the egg production [8].

Our results are in line with those of the existing literature since Omri et al. [11] stated that SP addition of 2.5% for a period of 6 weeks increased egg weight. According to Panaite et al. [20] poultry’s diet fortification with 2% of SP enhanced the weight of the generated eggs. In another study the authors described that the weight of the produced eggs recorded an increment after SP’s incorporation by 0.6% in laying hens’ diet [4].

The fortified group with SP5 displayed a higher Haugh unit content compared to the other investigated additions, including the control group, indicating an improved quality of the egg albumen. The said parameter forms a marker that reflects eggs’ quality, which is expressed by evaluating albumen’s height. The specific characteristic actually concerns the freshness and the overall quality of the egg [38,39,40]. Parisse [41] observed that SP addition in laying hens’ diet enhanced the Haugh unit score. Another study demonstrated that SP’s presence in a ratio of 5 g/Kg recorded a higher Haugh unit value [4]. The improved Haugh unit that was observed in the group of 5% *w*/*w* addition may enhance the protein content, as well as the structural integrity of the egg albumen, exhibiting thus a positive impact on the overall egg quality [4].

In the present work, the generated treatments were assessed for their total carotenoids, a parameter which is closely connected with yolk color. The derived results confirmed that the fortification with 5% *w*/*w* resulted in the highest concentration compared to the other samples. This parameter could presumably be a potent indicator of an improved egg quality, with potential health-promoting effects for consumers.

Yolk color is a significant parameter that was examined during the feeding trial. According to the obtained results yolk color was positively associated with the increasing levels of SP addition, indicating a dose-dependent effect. Specifically, the treatments with SP2 and SP5 displayed an enhanced yolk color with the SP5 addition, recording the highest score. This fact could be justified by carotenoid accumulation and more specifically beta-carotene in the yolk, thus resulting in an enhanced color [21,22]. Our results are in line with those of other studies since the authors stated that the implementation of SP has a direct effect on yolk color [23,42]. Yunitasari et al. [43] observed that SP addition of 1.5%, 2%, and 2.5% to the basal diet recorded gradual yolk intensification by 10.55, 11.43, and 11.66, respectively, compared with the control. Zahroojian et al. [44] stated that SP inclusion in a range of 1.5–2.5% led to a significant enhanced yolk color, with SP addition of 2.5% producing a pigmentation effect comparable to that of synthetic pigments.

Eggshell strength and quality are two important parameters that are crucial for minimizing a potential risk of breakage during handling and transportation. The present work revealed that gradual SP addition to laying hens diet improved the eggshell strength significantly, with the SP5 inclusion recording the highest value. This observation could be rationalized by SP’s rich chemical composition, which is highlighted by the presence of nutrients, namely calcium and phosphorus. The latter has the ability to improve the eggshell characteristics substantially by enhancing their quality and promoting strong shell formation [8]. Additionally, the present study examined the effect of Spirulina supplementation on the oxidative profile of the employed laying hens during the experimental period. The obtained results revealed that the increasing levels of Spirulina’s presence exhibited a positive impact across all the investigated parameters. In specific, TBARS and CARBs’ concentration were found to be reduced among the experimental trials, with the SP5 addition displaying the lowest score. On the contrary, the latter’s presence recorded the highest values concerning TAC, GSH and CAT activity between the examined samples. The observed results support our hypothesis that SP supplementation has the potential to address the generated oxidative stress. The major antioxidant enzymes such as CAT and GSH act as a protective agents against oxidative stress and enhance the overall immune status of poultry immune system [45]. CAT forms a crucial antioxidant enzyme which is responsible for the reduction in oxidative stress by decomposing hydrogen peroxide [46]. Our results are consistent with those of Park et al. [47], who stated that the administration of SP in broilers showed an increment in GPx and SOD enzymes, which was presumably justified by the high presence of antioxidants in SP’s composition. Additionally, Wu et al. [48] stated that SP supplementation exhibited a strong antioxidant capacity probably due to the higher content of phenolic compounds. According to another study, oxidative stress was found to be improved after the inclusion of 9% *w*/*w* SP in laying hens’ basal diet. The latter fact could be presumably rationalized by the presence of several bioactive molecules in SP’s chemical profile, such as flavonoids and polyphenols, and other free-radical scavengers, including α-tocopherol, ascorbic acid, β-carotene, and selenium [46,47].

According to Ghanouni-Bostanabad et al. [48], the addition of Spirulina of 5 g/kg had a positive effect on the antioxidant mechanism since the antioxidant enzymes CAT, SOD and GST, as well as non-enzymatic antioxidant GSH, were found to be increased. On the other hand, the latter contributed to the reduction in the MDA’s presence, which is a representative biomarker of lipid peroxidation. These modifications can be justified by SP’s antioxidant profile and therefore exhibit the potential to support the hypothesis that improved redox status can lead to a better performance and egg quality. Tufarelli et al. [23] reported that SP inclusion of 2% increased the total superoxide dismutase content, as well as enhanced total antioxidant capacity. In another study where the role of dietary SP supplementation in relieving the negative impacts of heat stress (HS) on redox status was investigated, it was reported that the addition of 9% reduced MDA and ceruloplasmin concentration with a parallel increment of GSH and TAC levels in laying hens’ blood. The ability of SP to maintain the redox balance may be a result of the bioactive molecules that are contained in SP’s composition, including flavonoids and polyphenols, as well as other free radical scavengers such as α-tocopherol, ascorbic acid, β-carotene, and selenium [49].

Mechanistically, phycocyanin, the major blue pigment protein of SP, is a potent free-radical scavenger that can directly neutralize reactive oxygen species and reduce lipid peroxidation (reflected here by the decline in TBARS), while also being reported to up-regulate endogenous antioxidant enzyme systems, consistent with the increase in CAT and GSH observed in the SP2 and SP5 groups [18]. In parallel, SP-derived carotenoids and phenolic compounds can contribute to redox homeostasis both through direct radical-scavenging activity and by supporting the cellular antioxidant defense network, which is consistent with the concurrent rise in TAC [48,49]. Because the higher-dose diets (SP2, SP5) also supplied more total protein, essential amino acids and energy, part of the improved redox status at these levels may additionally reflect an improved overall nutritional/metabolic status of the hens, rather than a purely antioxidant-specific mechanism of SP.

## 5. Limitations of the Study

The “on top” addition of SP to the basal diet of laying hens represents a significant limitation since the developed experimental diets were not reformulated in order to be iso-nitrogenous and iso-caloric. Consequently, the nutritional composition of the used diets were different concerning SP’s inclusion especially in the SP2 and SP5 groups. Therefore, the observed biological effects in the higher additions can be justified not only by SP’s rich bioactive profile but also by the potential synergistic effect of the enhanced nutritional composition of the employed diets. Additionally, it must be noted that SP’s metabolizable energy is approximately 3000 kcal/kg for poultry. Hence, the “on top” addition, especially in higher proportions such as 2 and 5%, enhanced the energy contribution to the respective diets. Therefore, the potential contribution of this additional energy to the observed responses cannot be completely excluded. Furthermore, the present study does not include an economic or cost–benefit analysis of SP supplementation, which would be an essential tool for ensuring the sustainable implementation of higher inclusion levels. Future studies should formulate iso-nitrogenous and iso-caloric diets and incorporate economic evaluations to determine the practical applicability of different SP inclusion levels under commercial production conditions.

## 6. Conclusions

The present study evaluated the effect of SP addition in various ratios in the basal diet of laying hens in relation to the internal and external egg quality characteristics, physicochemical profile and the oxidative stress in their blood. The obtained results revealed that the addition by 5% *w*/*w* exhibited a positive impact since the specific fortification enhanced the nutritional value of the generated feedstuff and improved latter parameters (egg weight, yolk intensity color, oxidative stress). However, despite the fact that the higher SP addition led to better results further studies must be conducted in the future, which will investigate whether these improvements are a result of the bioactive profile or of the additional nutrients that are supplied due to higher inclusion levels. Overall, the utilization of microalgae forms an intriguing approach to the improvement of the performance, physiology, and health of laying hens, with a parallel contribution to the development of more sustainable viable feed ingredients for poultry production systems.

## Figures and Tables

**Table 2:** Chemical composition of the commercial *Spirulina platensis* powder.

Parameter	Value
Crude protein (%)	67.1
Lipids (%)	8.2
Carbohydrates (%)	11.6
Dietary fiber (%)	3.8
Ash (%)	8.05
Phycocyanin (g/100 g)	2.36
β-carotene (mg/kg)	143
Vitamin E (mg/kg)	61.9
Estimated metabolizable energy for laying hens (kcal/kg)	3000

**Table 3:** Baseline egg quality characteristics before the experimental dietary treatments.

Parameter	Values
Weight (g)	47.45 ± 4.09
Height (mm)	51.90 ± 3.27
Diameter (mm)	40.44 ± 1.94
Yolk weight (g)	8.65 ± 1.12
Yolk height (mm)	11.39 ± 1.50
Yolk diameter (mm)	33.68 ± 1.86
Yolk moisture (%)	51.34 ± 1.52
Yolk pH	7.10
Yolk color (15-point color scale by using a DSM fan)	11.00
Albumen weight (g)	28.32 ± 4.78
Albumen height (mm)	5.83 ± 1.07
Albumen moisture (%)	86.92 ± 0.68
Albumen pH	9.73
Carotenoids (mg/g per dry Yolk)	64.83 ± 5.07
Eggshell thickness (mm)	0.40 ± 0.08
Eggshell weight (g)	4.11 ± 0.76

**Table 4:** The effect of *Spirulina platensis* on egg quality parameters at Day 60 (*n* = 15).

Parameters	Control	0.1% (*w*/*w*)	0.2% (*w*/*w*)	2% (*w*/*w*)	5% (*w*/*w*)
**External Egg Quality**					
Weight (g)	65.87 ± 3.22 ^a^	66.44 ± 4.55 ^a^	65.60 ± 4.33 ^a^	65.17 ± 2.43 ^a^	73.15 ± 3.55 ^b^
Height (mm)	56.37 ± 1.75 ^a^	58.42 ± 4.32 ^ab^	56.36 ± 2.18 ^a^	56.59 ± 1.33 ^a^	60.65 ± 1.66 ^b^
Diameter (mm)	45.78 ± 0.53 ^a^	45.61 ± 0.95 ^a^	45.57 ± 0.98 ^a^	45.41 ± 0.65 ^a^	50.12 ± 0.63 ^b^
Eggshell thickness (mm)	0.44 ± 0.02 ^a^	0.49 ± 0.05 ^a^	0.43 ± 0.03 ^a^	0.43 ± 0.06 ^a^	0.69 ± 0.02 ^b^
Eggshell weight (g)	6.33 ± 0.36 ^a^	6.66 ± 0.54 ^a^	6.23 ± 0.48 ^a^	6.12 ± 0.94 ^a^	8.49 ± 0.51 ^b^
**Internal Egg Quality**					
Yolk weight (g)	14.74 ± 0.37 ^a^	14.29 ± 0.64 ^a^	13.68 ± 0.91 ^a^	15.14 ± 0.80 ^a^	18.78 ± 0.86 ^a^
Yolk height (mm)	14.24 ± 1.50 ^a^	14.63 ± 1.29 ^a^	13.90 ± 0.57 ^a^	14.64 ± 0.82 ^a^	17.73 ± 1.23 ^b^
Yolk diameter (mm)	37.71 ± 1.01 ^a^	36.13 ± 0.76 ^a^	37.22 ± 1.37 ^a^	37.57 ± 1.95 ^a^	40.48 ± 1.15 ^b^
Yolk moisture (%)	47.24 ± 0.21 ^a^	47.69 ± 0.08 ^a^	46.85 ± 0.18 ^a^	47.54 ± 0.11 ^a^	48.07 ± 0.09 ^a^
Yolk pH	6.65 ^a^	6.70 ^a^	6.75 ^a^	6.70 ^a^	6.65 ^a^
Yolk color (15-point color scale by using a DSM fan)	11.38 ^a^	11.79 ^a^	12.34 ^a^	13.25 ^ab^	14.20 ^b^
Yolk Index	0.34 ± 0.04 ^a^	0.36 ± 0.04 ^a^	0.37 ± 0.02 ^a^	0.39 ± 0.03 ^a^	0.51 ± 0.04 ^b^
Albumen weight (g)	41.34 ± 3.07 ^a^	43.03 ± 3.86 ^a^	42.29 ± 3.81 ^a^	40.12 ± 1.89 ^a^	46.01 ± 3.43 ^b^
Albumen height (mm)	7.12 ± 1.54 ^a^	6.95 ± 1.68 ^a^	7.99 ± 1.44 ^a^	7.04 ± 1.44 ^a^	10.46 ± 1.00 ^b^
Albumen moisture (%)	86.35 ± 0.49 ^a^	86.08 ± 0.84 ^a^	86.28 ± 0.76 ^a^	86.43 ± 0.66 ^a^	85.84 ± 1.43 ^a^
Albumen pH	9.30 ^a^	9.20 ^a^	9.05 ^a^	9.30 ^a^	9.40 ^a^
Haugh units	62.09 ± 0.61 ^a^	64.80 ± 0.85 ^a^	65.14 ± 0.81 ^a^	67.22 ± 0.46 ^a^	76.22 ± 0.67 ^b^
Egg strength (g)	3.84 ± 0.10 ^a^	3.89 ± 0.22 ^a^	3.68 ± 0.19 ^a^	3.69 ± 0.18 ^a^	5.09 ± 0.17 ^b^

Different superscripts (a,b) in the same line indicate the presence of statistical significance (*p* < 0.05). Superscript letters are compared horizontally, within each parameter (row), across the five dietary treatments. Data are means ± SD of five replicate cages per treatment.

**Table 5:** Eggs’ physicochemical profile of laying hens fed with fortified ration with *Spirulina platensis* (*n* = 15).

Parameters	Control	0.1% (*w*/*w*)	0.2% (*w*/*w*)	2% (*w*/*w*)	5% (*w*/*w*)
Carotenoids (mg/g per dry Yolk)	43.98 ± 0.75 ^a^	70.09 ± 3.40 ^b^	76.10 ± 1.75 ^b^	120.84 ± 2.57 ^c^	172.83 ± 3.90 ^d^
Yolk proteins (%)	33.57 ± 1.73 ^a^	33.66 ± 0.87 ^a^	33.20 ± 0.87 ^a^	33.47 ± 1.41 ^a^	40.84 ± 0.65 ^b^
Albumen proteins (%)	85.87 ± 1.92 ^a^	84.09 ± 1.44 ^a^	84.22 ± 1.29 ^a^	90.77 ± 2.68 ^b^	93.39 ± 2.14 ^b^
Eggshell ash content (%)	92.91 ± 0.77 ^a^	92.83 ± 0.59 ^a^	93.87 ± 0.27 ^a^	92.15 ± 3.74 ^a^	92.88 ± 1.55 ^a^

Different superscripts (a,b,c,d) in the same line indicate the presence of statistical significance (*p* < 0.05). Superscript letters are compared horizontally, within each parameter (row), across the five dietary treatments. Data are means ± SD of five replicate cages per treatment.

**Table 6:** Egg fatty acid composition in the experimental diets (*n* = 15).

Fatty Acids	Control	0.1% *w*/*w*	0.2% *w*/*w*	2% *w*/*w*	5% *w*/*w*
**C14:0 myristic acid**	0.43 ± 0.06 ^a^	0.41 ± 0.01 ^a^	0.39 ± 0.01 ^a^	0.39 ± 0.04 ^a^	0.39 ± 0.01 ^a^
**C14:1 myristoleic acid. Cis-9**	0.07 ± 0.01 ^a^	0.08 ± 0.02 ^a^	0.08 ± 0.02 ^a^	0.07 ± 0.01 ^a^	0.07 ± 0.02 ^a^
**C15:0 pentadecanoic acid**	0.10 ± 0.02 ^a^	0.09 ± 0.01 ^a^	0.10 ± 0.01 ^a^	0.08 ± 0.01 ^a^	0.09 ± 0.02 ^a^
**C16:0 palmitic acid**	26.53 ± 0.86 ^a^	26.92 ± 1.06 ^a^	27.19 ± 0.04 ^a^	26.29 ± 0.61 ^a^	27.91 ± 0.31 ^a^
**C16:1 palmitoleic acid**	2.43 ± 0.65 ^a^	2.69 ± 0.19 ^a^	2.82 ± 0.15 ^a^	2.53 ± 0.24 ^a^	2.91 ± 0.30 ^a^
**C17:0 heptadecanoic acid**	0.30 ± 0.03 ^a^	0.23 ± 0.04 ^b^	0.27 ± 0.01 ^ab^	0.28 ± 0.02 ^a^	0.33 ± 0.02 ^a^
**C18:0 stearic acid**	7.87 ± 0.85 ^a^	9.22 ± 0.91 ^b^	8.64 ± 0.35 ^a^	9.27 ± 0.02 ^b^	8.84 ± 0.51 ^b^
**C18:1 oleic acid. Cis-9**	38.15 ± 1.03 ^ab^	39.82 ± 0.69 ^ab^	38.62 ± 1.01 ^ab^	40.29 ± 0.69 ^b^	37.43 ± 1.37 ^a^
**C18:2 linoleic acid. Cis-6**	19.33 ± 0.69 ^a^	15.76 ± 2.94 ^b^	17.26 ± 0.47 ^ab^	16.08 ± 1.48 ^b^	17.30 ± 0.60 ^ab^
**C18:3 gama-linolenic acid. Cis-6**	0.16 ± 0.02 ^a^	0.15 ± 0.01 ^a^	0.18 ± 0.01 ^a^	0.16 ± 0.02 ^a^	0.22 ± 0.05 ^b^
**C18:3 a-linolenic acid. Cis-3**	0.64 ± 0.11 ^a^	0.50 ± 0.16 ^b^	0.57 ± 0.05 ^a^	0.52 ± 0.05 ^b^	0.55 ± 0.03 ^ab^
**C20:1 eicosenoic acid. Cis-9**	0.25 ± 0.03 ^a^	0.26 ± 0.02 ^a^	0.25 ± 0.02 ^a^	0.24 ± 0.02 ^a^	0.20 ± 0.01 ^b^
**C20:2 eicosadienoic acid**	0.21 ± 0.01 ^a^	0.18 ± 0.03 ^b^	0.20 ± 0.02 ^a^	0.18 ± 0.02 ^b^	0.18 ± 0.01 ^b^
**C20:3 homo-gama-linolenic acid. Cis-6**	0.17 ± 0.06 ^a^	0.17 ± 0.01 ^a^	0.17 ± 0.01 ^a^	0.16 ± 0.01 ^a^	0.19 ± 0.02 ^a^
**C20:4 arachidonic acid. Cis-6**	2.09 ± 0.12 ^a^	2.26 ± 0.25 ^a^	2.25 ± 0.03 ^a^	2.35 ± 0.16 ^a^	2.24 ± 0.13 ^a^
**C22:2 docosadienoic acid**	0.52 ± 0.07 ^a^	0.56 ± 0.13 ^a^	0.26 ± 0.09 ^b^	0.36 ± 0.14 ^c^	0.42 ± 0.02 ^c^
**C22:6 DHA. Cis-3**	0.77 ± 0.04 ^a^	0.71 ± 0.08 ^a^	0.77 ± 0.01 ^a^	0.76 ± 0.01 ^a^	0.72 ± 0.09 ^a^
**∑MUFA**	40.89 ± 0.86 ^ab^	42.84 ± 0.90 ^ab^	41.76 ± 1.17 ^ab^	43.12 ± 0.96 ^b^	40.62 ± 1.69 ^a^
**∑PUFA**	23.89 ± 1.03 ^a^	20.29 ± 1.60 ^b^	21.66 ± 0.69 ^b^	20.56 ± 1.87 ^b^	21.82 ± 0.95 ^b^

∑MUFA = sum of total monounsaturated fatty acids, ∑PUFA = sum of total polyunsaturated fatty acids. Different superscripts (a,b,c) in the same line indicate the presence of statistical significance (*p* < 0.05). Superscript letters are compared horizontally, within each parameter (row), across the five dietary treatments. Data are means ± SD of five replicate cages per treatment.

**Table 7:** Effect of SP addition on the oxidative profile of laying hens (n = 15).

Parameters	Control	0.1% (*w*/*w*)	0.2% (*w*/*w*)	2% (*w*/*w*)	5% (*w*/*w*)
TBARS (μmol/L plasma)	16.13 ± 0.61 ^a^	15.09 ± 0.33 ^a^	13.91 ± 0.25 ^b^	12.56 ± 0.46 ^b^	10.34 ± 0.27 ^c^
CARBs (nmol/mg protein)	0.95 ± 0.11 ^a^	0.88 ± 0.14 ^a^	0.81 ± 0.09 ^b^	0.69 ± 0.17 ^c^	0.54 ± 0.08 ^d^
TAC (mmol DPPH/L plasma)	0.48 ± 0.12 ^a^	0.59 ± 0.10 ^b^	0.75 ± 0.07 ^c^	0.96 ± 0.11 ^d^	1.23 ± 0.08 ^e^
GSH (μmol/g Hb)	18.20 ± 0.46 ^a^	19.62 ± 0.87 ^ab^	20.41 ± 0.74 ^b^	26.93 ± 0.81 ^c^	31.19 ± 0.73 ^d^
CAT (U/mg Hb)	13.21 ± 0.25 ^a^	15.14 ± 0.17 ^b^	16.11 ± 0.36 ^b^	21.64 ± 0.44 ^c^	25.69 ± 0.79 ^d^

Different superscripts (a,b,c,d,e) in the same line indicate the presence of statistical significance (*p* < 0.05). Superscript letters are compared horizontally, within each parameter (row), across the five dietary treatments. Data are means ± SD of five replicate cages per treatment.

## Data Availability

The original contributions presented in this study are included in the article. Further inquiries can be directed to the corresponding author.

## Abbreviations

The following abbreviations are used in this manuscript:

CARBs	Protein carbonyls
MDA	Malondialdehyde
TBARS	Thiobarbituric acid reactive substances
CAT	Catalase activity
GSH	Reduced glutathione
TAC	Total antioxidant content
SP	*Spirulina platensis*
